# Electroencephalogram Analysis of Magnetic Stimulation at Different Acupoints

**DOI:** 10.3389/fnins.2022.848308

**Published:** 2022-04-05

**Authors:** Ning Yin, Ao-Xiang Wang, Hai-Li Wang

**Affiliations:** ^1^State Key Laboratory of Reliability and Intelligence of Electrical Equipment, Hebei University of Technology, Tianjin, China; ^2^Tianjin Key Laboratory of Bioelectromagnetic Technology and Intelligent Health, Hebei University of Technology, Tianjin, China

**Keywords:** magnetic stimulation, acupoint, EEG, PLV, time-frequency analysis

## Abstract

Magnetic stimulation has some similarities with acupuncture, and it has broad application prospects because of its non-invasiveness and easy quantification. This paper combines magnetic stimulation technology with electroencephalography to analyze the time-frequency and the brain functional network results elicited by magnetic stimulation at different acupoints. This paper hopes to observe the different effects of stimulating different acupoints on the brain from the perspective of EEG. The EEG signals during magnetic stimulation at ST36, ST40, and GB37 were recorded, respectively. The time-frequency results showed that the magnetic stimulation at ST36 and ST40 on the Foot Yangming Stomach Meridian increased the energy in the left parietal lobe and the right central region, and the energy increased mainly in the theta and alpha bands. However, during the magnetic stimulation at GB37 on the Foot Shaoyang Gallbladder Meridian, the energy in the central region and the frontal lobe increased, and the energy increased mainly in the delta, theta, and alpha bands. Moreover, the energy in the right parietal lobe decreased during magnetic stimulation at GB37. The results of brain functional network were also consistent with time-frequency results. The brain network connections of GB37 stimulation in the central region were significantly less than that of ST36 and ST40 (*p* < 0.01). In addition, the connections between central region and frontal lobe and the connections between central region and parietal lobe of GB37 stimulation were significantly different from that of ST36 and ST40 (*p* < 0.01). The above results indicate that ST36 and ST40 on the same meridian have similar effects on the brain, while GB37 on the other meridian has completely different effects from ST36 and ST40. The results of this paper explain the reason why stimulating ST36 and ST40 can treat similar diseases from the perspective of EEG, and also explain that stimulating GB37 has significantly different effects on the brain from that of ST36 and ST40.

## Introduction

Acupuncture, which originated in China, has a history of more than 2,000 years and is a treasure of human civilization. By stimulating different acupoints, acupuncture, and moxibustion can be widely used in the treatment of heart disease (Crook et al., 2001), schizophrenia (Dai et al., 2019), depression (Fuchs et al., 2002), anxiety (Geng et al., 2017), and obsessive-compulsive disorder (Gray et al., 2021). However, the imaging research on the mechanism of acupoint stimulation is still in its infancy, and there are some limitations in the research model and method, and the consistency of relevant research conclusions is low. Current medical theory holds that all the organs in an organism work in concert with each other in the brain (Heuvel and Pol, 2010). Therefore, modern medical theory links acupoint with brain, which provides a new research direction for the study of the mechanism of acupoint stimulation.

In recent years, the specific brain response to acupoint stimulation has attracted extensive attention. Based on different imaging techniques, it is possible to study the effect of acupuncture on the brain. Electroencephalogram (EEG) and functional magnetic resonance imaging (fMRI) are the main imaging methods for the acupoint research (Onton et al., 2005; Louise et al., 2009; Polikar et al., 2010; Ivor et al., 2013). EEG has the advantages of high temporal resolution, low cost, and ease of operation, and is expected to capture the transient changes caused by stimulation at a smaller time scale (Arroyo et al., 1992; Gevins, 1998).

Although the underlying mechanism of acupoint therapy is not clear, it is certain that acupuncture points are sensitive to various stimulus such as electromagnetic stimulation. Magnetic stimulation at acupoint has been studied since the 1970s (Jun et al., 2015). Magnetic stimulation technology has the advantages of being safe, painless, effective, and non-invasive. Also, it has a unique effect in the regulation of human nerve function, disease treatment, and rehabilitation physiotherapy, which has similar effects with traditional acupuncture and moxibustion. Combining magnetic stimulation with acupoints is a new example of combining modern medical technology with traditional Chinese medicine. It not only gives new content to the application of magnetic stimulation technology but also promotes the development of traditional Chinese medicine. Magnetic stimulation acupoints have always been our main research direction, and many good results have been obtained (Yu et al., 2014; Fu et al., 2017; Dai et al., 2019).

This paper collects EEG data of human brains. EEG signal is not a stationary signal; it is relatively complex and easy to be polluted by interference signals. The application of time-frequency analysis to EEG signals can realize the in-depth study of these non-stationary signals. In this paper, short-time Fourier method was used to analyze the EEG signals of magnetic stimulation at three acupoints.

As a non-linear signal, EEG is more suitable for non-linear analysis compared with linear methods. Synchronous likelihood is a non-linear analysis method that can simultaneously measure linear and non-linear synchronization between signal pairs, so it is widely used to construct brain networks. Brain network based on synchronous likelihood index can be used to study the effect of magnetic stimulation on functional connectivity of acupoints (Chen and Rappelsberger, 1994; Stam and Dijk, 2002; Blankenship et al., 2016; Jaime et al., 2016). Hongrui et al. (2011) found that acupuncture stimulation increased the complexity of the brain network by analyzing the non-linear indicators of EEG. Phase locking value (PLV), as a method to measure the phase difference between two time series signals, has been widely used in EEG analysis (Lachaux et al., 1999). Therefore, PLV was used to study the effect of acupuncture on phase synchronization of different electrodes.

Acupoint specificity is the basis and basis of guiding acupuncture point selection in clinic. Therefore, the corresponding relationship of “acupoint–brain” can be studied mainly from the aspects of single acupoint and non-acupoint, acupoint on the same and different meridian (Chen et al., 2010; Ping et al., 2010; Kwang-Ho et al., 2016a; Li et al., 2017). Kwang-Ho et al. (2016b) used high-frequency power spectrum analysis of EEG to evaluate the effects of acupoint and non-acupoint stimulation. Most researchers believe that there is a relatively specific relationship between the meridians, brain, and zang-fu organs, but there are still a few scholars who doubt it.

There are a large number of acupoints, and the diseases treated by different acupoints are obviously different. Therefore, this paper hopes to use magnetic stimulation technology to stimulate different acupoints to observe whether there are significant differences in the effects of stimulation on the brain. A number of previous studies have found that magnetic stimulation at acupoints has significantly greater effects on the brain than non-acupoint stimulation. For example, Geng (2010) found that magnetic stimulation at Shenmen point at 1 Hz can stimulate more cognitive activities than that at non-acupoints. Yin (2013) used the brain functional network for the first time to study the influence of magnetic stimulation at Neiguan point and found that the brain functional network had significant changes. Yuan et al. (2020) recruited 72 patients with chronic insomnia, including 36 patients in the control group who received standard behavioral cognitive therapy and 36 patients in the experimental group who received behavioral cognitive therapy combined with magnetic stimulation at Shenmen point. The recovery degree of patients was evaluated by sleep index, Beck Depression Scale, and Beck Anxiety Scale. The results showed that magnetic stimulation of Shenmen point could improve the cognitive function of patients with chronic insomnia, which was an effective treatment for chronic insomnia (Yuan et al., 2020).

In this paper, magnetic stimulation technology was used to stimulate at different acupoints and the EEG acquisition experiments were conducted. First, through time-frequency analysis of EEG signals, the influence of magnetic stimulation on the brain was observed in time domain and frequency domain. Then, the brain functional networks were constructed by using PLV and analyzed through graph theoretic features to observe the influence of magnetic stimulation at different acupoints.

## Materials and Methods

### Subjects and Experiment

Twenty healthy subjects, 12 men and 8 women, were recruited through advertising. All subjects are right-handed, between the ages of 21 and 25, with normal corrected vision and no history of infectious diseases or psychiatric disorders. The content of the experiment conforms to the requirements of the Declaration of Helsinki of the World Medical Association to carry out biomedical research with human subjects as subjects, and does not involve any ethical principles and moral issues. Before the experiment, the subjects were informed of all the operation steps and precautions of the acupoint magnetic stimulation experiment, and signed the informed consent.

**FIGURE 1:**
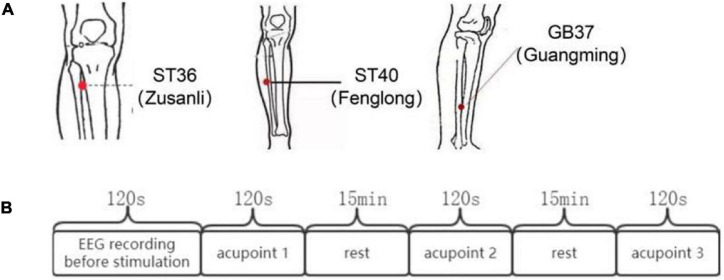
The locations of ST36, ST40, and GB37 and the flow chart of the experiment. **(A)** Locations of ST36, ST40, and GB37. **(B)** Experimental process.

In this experiment, three acupoints were selected, ST36, ST40, and GB37. The locations of the acupoints are shown in Figure 1A. ST36, ST40, and GB37 are all located in the lower leg. The stimulation method we use is unilateral stimulation of the right calf on the three points. This avoids the problem of unilateral and bilateral stimulation, and also avoids the problem of stimulating the upper and lower limbs. The three points we selected are all located on the front of the calf, and the stimulation in this paper is carried out from the front of the calf. Since there have been many previous studies on the specificity of magnetic stimulation at individual acupoint and non-acupoint, this experiment did not perform stimulation at non-acupoint, but mainly studied the differences between acupoints.

Rapid^2^ TMS system with Figure 8–shaped coil (Magstim Company Ltd., Whitland, United Kingdom) was used as the magnetic pulse generator. Stimulation frequency was 0.5 Hz. Intensity was 1.76 T, and 1.76 T is 80% of the maximum power output of the instrument. The center of Figure 8 coil was aligned to the acupoint to achieve precise stimulation. The Figure 8–shaped coil only has obvious magnetic stimulation intensity corresponding to the center point of the coil. Magnetic stimulation does not work through magnetic fields; it works by the same mechanism as electrical stimulation at the cellular level. The difference between the two is that electrical stimulation directly stimulates tissue cells by injecting current through electrodes, while magnetic stimulation uses changing pulse magnetic field to form induced current in human tissues through spatial coupling and stimulates tissue cells without contact. Magnetic field can basically have no attenuation through the skin, fat, bone, muscle, and other tissues, to deep tissues and cells (Barker, 1991). Studies have shown that under the same surface electric field value, compared with the surface electrode, the attenuation of the induced electric field generated by magnetic stimulation is much smaller when penetrating the head tissue, and the value of the induced electric field generated by magnetic stimulation at 40 mm is 10 times larger than that generated by the surface electric stimulation (Roth et al., 1991). In addition, magnetic stimulation does not have a very concentrated current density in the area of biological tissue, so subjects will not or only be slightly uncomfortable (Barker, 1991).

The experimental process is shown in Figure 1B. First, EEG data of subjects in the resting state of 120 s were collected synchronously. Then, the three different acupoints aforementioned were magnetically stimulated 120 s, respectively, and EEG data were collected synchronously. The order for different stimulation sessions was random across subjects to counteract any crossover effect.

### Electroencephalogram Signal Recording and Data Processing

EEG was recorded using a Quick-Cap 64-channel EEG cap connected to a Neuroscan Synamps (Neuroscan Ltd., Charlotte, NC, United States). The 64-channel EEG cap conforms to the international standard 10–20 system. AFZ was used as ground electrode. Neuroscan acquisition/edit software was used to collect the EEG data in DC mode at a sampling rate of 1 kHz. After the data collection, the data were preprocessed, including extract EEG segments, remove useless channels, channel locations, re-reference (average reference), 1–40 Hz filter, extract epochs (the resting state data was divided into 2-s segments, the stimulus state data were segmented according to 0.5 s before and 1 s after the mark), and reject data using ICA. After data preprocessing, the fast Fourier transform was processed for the subsequent time-frequency analysis. Also, PLV was used to construct brain functional network.

### Phase-Locking Value

Phase-locking value (PLV) is usually used to quantify the phase synchronization between narrowband signals, and its value represents the absolute value of the average phase difference between any two time signals. The definition formula of *PLV* is as follows:


(1) $$PLV_{t}=\frac{1}{N}\left|\sum_{n=1}^{N}exp\left(j\theta \left\{t,n\right\}\right)\right|$$

where *θ*(*t*, *n*) is the phase difference Φ_1_(*t*, *n*)−Φ_2_(*t*, *n*) and Φ(*t*, *n*) represents the instantaneous phase of the analytic signal. *N* is the point in time. *PLV* measures the intertrial variability of this phase difference at time *t*. If the phase difference varies little across the trials, the *PLV* is close to 1; otherwise, it is close to 0. The 60 × 60 adjacency matrix was obtained by calculating the PLV of each pair-wise EEG channel.

### Characteristic Parameters of Brain Network

#### Clustering Coefficient

Clustering coefficient is an effective index to evaluate the functional separation of network. It reflects the clustering degree of nodes in the brain network. The average clustering coefficient can be expressed as


(2) $$C=\frac{1}{N}\sum_{i=1}^{N}\frac{E_{i}}{M_{i}}=\frac{1}{N}\sum_{i=1}^{N}\frac{2E_{i}}{k_{i}\left(k_{i}-1\right)}$$

where *N* is the number of nodes, *k_i_* is the number of nodes connected to the node *i*, *E_i_* is the total number of actual connection edges between the nodes connected to the node *i*, and *M_i_* is the maximum value of possible connection edges between the nodes connected to the node *i*.

#### Characteristic Path Length

The connection path between nodes may be directly connected or may form a common connection through other nodes. The shortest path length describes the optimal transmission path of functional information interaction between two nodes. The average value of the shortest path length of all nodes, namely the characteristic path length, can be used to evaluate the efficiency of global information transmission in the network. High feature average shortest path length indicates slow information transfer in the network. The calculation method of characteristic path length *L* is as follows:


(3) $$L=\frac{1}{N\left(N-1\right)}\sum_{i\neq j}d_{ij}$$

where *d*_*ij*_ is the shortest path length between node *i* and node *j*.

#### Degree of Node

The degree of a node is defined as the number of nodes directly connected to the node. It mainly describes the statistical characteristics of the connection edges between nodes and reflects the evolution characteristics of complex networks. The average degree is expressed as


(4) $$D=\frac{1}{N}\sum_{i=1}^{N}D_{i},$$

where *N* is the number of nodes and *D_i_* is the degree of node *i*.

### Statistical Analysis

One-way ANOVA was used to determine whether there were statistical differences among the three acupuncture points of magnetic stimulation. The Bonferroni method was used for multiple comparative post-analysis of PLV and network characteristics of the three states, and all *p*-values were corrected for multiple times. The indices calculated from the mean and variance of state parameters extracted from graph theory analysis show the significance of differences between groups, where *p* < 0.05 (*), *p* < 0.01 (^**^), and *p* < 0.001 (^***^) represent the significance level of statistical analysis. Since the ANOVA requires that the independent sample must meet the normality, Fisher-Z transformation is used in this paper to improve the normality of the independent sample.

## Results

### Time-Frequency Analysis Results

**FIGURE 2:**
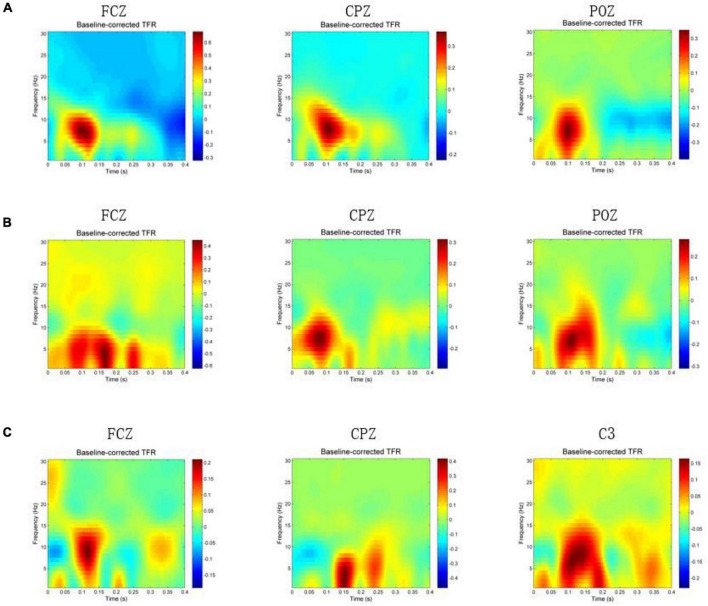
Time-frequency results when magnetic stimulation is carried out at acupoints. In the figure, *x*-axis means time (s), *y*-axis is frequency (Hz), and the color block represents the energy (V^2^). **(A)** Time-frequency results of FCZ, CPZ, and POZ electrodes during magnetic stimulation at ST36. **(B)** Time-frequency results of FCZ, CPZ, and POZ electrodes during magnetic stimulation at ST40. **(C)** Time-frequency results of FCZ, CPZ, and C3 electrodes during magnetic stimulation at GB37.

A participant was excluded because of bad data, and the subsequent analysis was carried out on the remaining 19 participants. Figure 2 shows the group average time-frequency analysis results during magnetic stimulation at ST36, ST40, and GB37. All the figures show the energy of the magnetic stimulation subtracted from the baseline energy, directly showing the increase or decrease of energy after the stimulus. Figure 2A shows the time-frequency results of FCZ, CPZ, and POZ electrodes during magnetic stimulation at ST36. Figure 2B shows the time-frequency results of FCZ, CPZ, and POZ electrodes during magnetic stimulation at ST40. Figure 2C shows the time-frequency results of FCZ, CPZ, and C3 electrodes during magnetic stimulation at GB37. Specific values are shown in Table 1. It can be seen from Figure 2A that the energy between 50–150 ms and 4–13 Hz (theta and alpha) increases significantly at the electrodes of FCZ, CPZ, and POZ during magnetic stimulation at ST36. At the same time, after statistics, it was found that during magnetic stimulation at ST36, all significant energy changes occurred in 50–150 ms and 4–13 Hz (theta and alpha).

**TABLE 1:** Energy values of each electrode before and after stimulation in Figure 2.

	Average energy before stimulation (μV^2^)	Maximum energy after stimulation (μV^2^)	Percentage of energy increase (%)
FCZ of ST36	0.60	1.20	100
CPZ of ST36	0.40	0.70	75
POZ of ST36	0.36	0.60	67
FCZ of ST40	0.60	1.00	67
CPZ of ST40	0.38	0.70	84
POZ of ST40	0.44	0.70	59
FCZ of GB37	0.40	0.60	50
CPZ of GB37	0.70	1.10	57
C3 of GB37	0.35	0.53	51

As can be seen from Figure 2B, the energy of the FCZ electrode increases at 50–200 ms, 0–10 Hz and 220–270 ms, 0–7 Hz. Also, the energy increase of CPZ electrode during the magnetic stimulation at ST40 mainly occurs between 50–150 ms and 4–13 Hz (theta and alpha), which is consistent with the magnetic stimulation at ST36. POZ electrode has increased energy between 50–200 ms and 0–15 Hz. Statistically, it was found that the energy of electrode in the frontal lobe increased at the same time and frequency band as the FCZ electrode, and the energy of electrode in the parietal lobe increased at the same time and frequency band as the POZ electrode.

As shown in Figure 2C, different from the other two groups, the energy increase at the CPZ electrode is mainly between 120–180 ms and 0–8 Hz (delta and theta), and the energy also increases between 200–250 ms and 0–10 Hz. Figure 2C also shows the time-frequency results of C3 and FCZ electrodes during magnetic stimulation at GB37. It can also be seen that the energy of C3 electrode increases at 50–200 ms and 0–13 Hz, and the energy of FCZ electrode increases at 70–150 ms and 0–15 Hz. According to statistics, during magnetic stimulation at GB37, the energy increase area of central region was mainly consistent with that of C3 electrode, and few electrodes of central region results were consistent with CPZ. The energy increase area of frontal lobe was consistent with that of FCZ electrode.

This paper observed the time-frequency results of each electrode one by one. The electrodes with energy increased and decreased are shown in Table 2. It can be seen that the electrodes with increased energy during magnetic stimulation at ST36 and ST40 are mainly in the left parietal lobe, right central region, and left frontal lobe. When magnetically stimulated at GB37, the electrodes with energy increased are mainly in the central region and frontal lobe, while the electrodes with energy decreased are in the right parietal lobe.

**TABLE 2:** Electrodes whose energy changes during magnetic stimulation at three acupoints.

	Energy-raising electrodes	Energy-reducing electrodes
ST36	O1, POZ, PO3, PO5, PO7, P3, P5, P7, CPZ, C6, C4, C2, CZ, C1, FC4, FC2, FCZ, F4	None
ST40	OZ, O1, POZ, PO3, PO5, PO7, CPZ, C4, C2, CZ, C1, FC4, FC2, FCZ, FC1	None
GB37	CPZ, C2, CZ, C1, C5, FC2, FCZ, FC1, FC5, F5, F7	P4, P6, P8, PO4, PO6, PO8, O2, OZ

### Analysis of Brain Functional Networks

**FIGURE 3:**
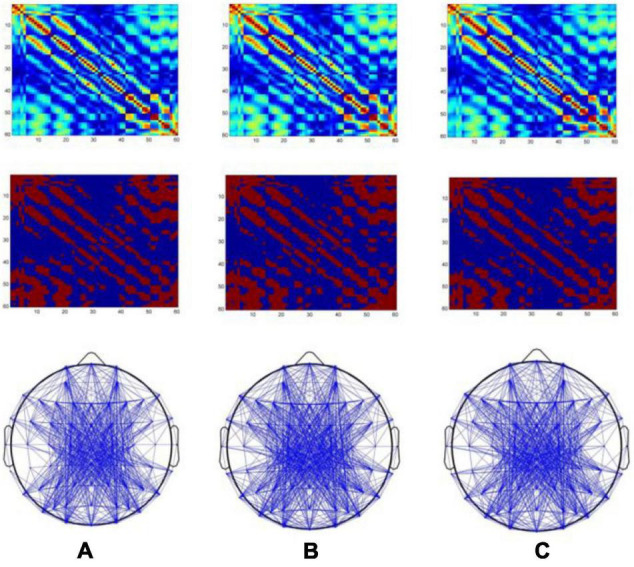
Brain functional networks when magnetic stimulation is carried out at acupoints. The *x*- and *y*-axes of the first and second rows of the figure represent electrodes (1–60). **(A–C)** Results of magnetic stimulation at ST36, ST40, and GB37, respectively.

The PLV between EEG channels in each subject was calculated to quantify the functional connectivity between different EEG channels. The EEG signals ranging from 0.5 to 30 Hz were used. In this study, the locations of 60 electrodes of scalp were selected as network nodes. PLV was applied to investigate synchronization dynamics of magnetic stimulation at acupoints. Figures 3A–C shows the results of magnetic stimulation at ST36, ST40, and GB37, respectively. The first row of Figure 3 is the results of PLV matrix. An appropriate threshold of 0.39 was set to construct the binarization matrix, as shown in the second row of Figure 3. The construction of the brain functional networks based on the binarization matrix are shown in the third row of Figure 3.

**FIGURE 4:**
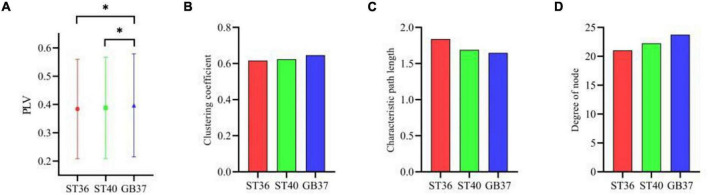
The graph theoretic features of three stimulation states. **(A)** Mean PLVs of the whole brain during magnetic stimulation at the acupoints. **(B–D)** Clustering coefficient, characteristic path length, and node degree during magnetic stimulation at the acupoints. *p < 0.05.

Furthermore, three graph theoretic features were extracted from the brain functional networks, including clustering coefficient, characteristic path length, and the degree of node. Figure 4A shows the mean PLV values of the whole brain during magnetic stimulation at the acupoints. As can be seen in Figure 4A, there are significant differences (*p* < 0.05) between the mean PLVs of ST36 and GB37, as well as between ST40 and GB37. As shown in Figures 4B–D, GB37 stimulation has a larger clustering coefficient, shorter characteristic path length, and higher node degree compared with ST36 and ST40, indicating that GB37 stimulation has a higher degree of global brain aggregation, higher global information transmission efficiency, and more active brain functional network.

**FIGURE 5:**
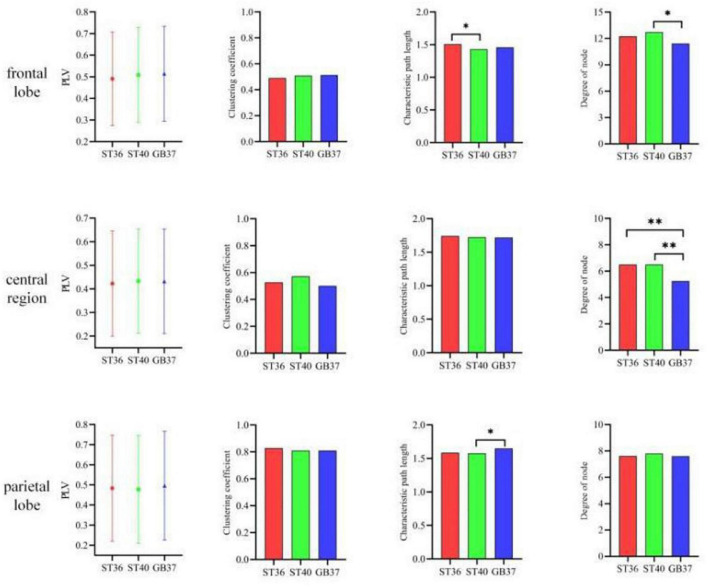
The graph theoretic features in the frontal lobe, central region, and parietal lobe. *p < 0.05 and ^**^p < 0.01.

To observe in which brain regions the differences between the three stimulation states occur, we made statistics on the graph theoretic features of the frontal lobe, central region, and parietal lobe of each state, and the results are shown in Figure 5. It can be seen in the second row and fourth column of Figure 5 that the degrees of node in the central region of ST36 and ST40 stimulation are basically the same and significantly larger (*p* < 0.01) than GB37. It indicates that ST36 and ST40 stimulation have more brain functional network connections than GB37. As can be seen from the first and third rows of Figure 5, there is no obvious difference (*p* > 0.01) between ST36, ST40, and GB37 in the frontal and parietal lobes.

**FIGURE 6:**
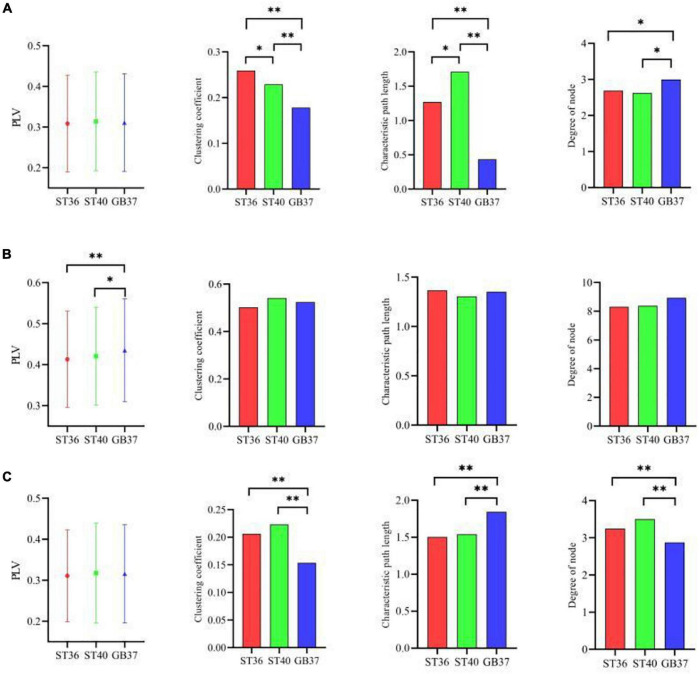
Graph theoretic features of the functional connections between brain regions. **(A)** Graph theoretic features between the frontal lobe and the central region. **(B)** Results between the frontal lobe and parietal lobe. **(C)** Results between the central region and parietal lobe. *p < 0.05 and ^**^p < 0.01.

In addition to the differences within the brain regions, there are also differences in the connections between brain regions. Figure 6 shows the graph theoretic features between brain regions of ST36, ST40, and GB37 stimulation. Figure 6A represents the graph theoretic features between the frontal lobe and the central region, Figure 6B shows the results between the frontal lobe and parietal lobe, and Figure 6C shows the results between the central region and parietal lobe. As shown in Figure 6A, the characteristic path length is significantly shorter (*p* < 0.01), and the node degree is significantly larger (*p* < 0.05) in the group of GB37 stimulation. It indicated that the brain functional connections between the frontal lobe and the central region of GB37 stimulation were significantly more than that of ST36 and ST40. As can be seen from Figure 6B, there was no significant difference in connections between frontal and parietal lobes of ST36, ST40, and GB37. As can be seen from Figure 6C, the clustering coefficient of GB37 stimulation is significantly smaller (*p* < 0.01), the path length is significantly larger (*p* < 0.01), and the node degree is significantly smaller (*p* < 0.01). It indicated that compared with ST36 and ST40 stimulation, GB37 stimulation had significantly fewer brain functional connections between central region and frontal lobe.

## Discussion

ST36 is located on the Foot-Yangming Stomach Meridian in Chinese medicine. A large number of clinical studies have shown that ST36 has significant effects on various symptoms such as sleep disorder relief, stress control, edema suppression, and inflammation reduction (Barker, 1991). Acupuncture at ST36 has a regulatory effect on brain activity (Roth et al., 1991). ST40 and ST36 are located on the same meridian, and these two acupoints are usually used together to treat diseases in many clinical cases (Haitao et al., 2019). Chang et al. (2004) studied the regulation effect of acupuncture at ST36 on the brain through fMRI, and found that the insula, amygdala, and anterior cingulate gyrus were activated, while the sensory cortex and frontal lobe were inhibited. Tan et al. (2017) compared the difference between acupuncture at ST40 and non-acupoint through fMRI, and found that acupuncture at ST40 has a significant enhancement effect on the right inferior frontal gyrus, parietal lobule, and left inferior parietal lobule, and has a significant weakening effect on the thalamus, anterior cingulate gyrus, middle frontal gyrus, posterior cerebellar lobe, and the left parahippocampal gyrus, insula, and anterior cerebellar lobe. Relevant studies have shown that acupuncture at ST36 and ST40 can activate brain regions related to cognition and pain processing. Wu et al. (2008) used fMRI to observe the difference between acupuncture at ST36 and ST40, and found that the brain activities were increased in the left parahippocampal gyrus, left middle temporal gyrus, left superior temporal gyrus, right superior temporal gyrus, and right superior limbic gyrus during acupuncture at ST36, and the brain activities were also increased to a certain extent in the left superior temporal gyrus, left middle temporal gyrus, right superior temporal gyrus, right cingulate gyrus, right medial frontal gyrus, and right paracentral lobule when ST40 was acupunctured. It is found that acupuncture at different points located on the same meridian can produce similar but different physiological effects (Feng et al., 2011).

Through our results, we found that during magnetic stimulation at ST36 and ST40 located on the same meridian, the time-frequency analysis results were basically consistent. As shown in Table 1, the energy increased mainly in the left parietal lobe and the right central region, and the energy of a few electrodes in the frontal lobe and occipital lobe also increased. Moreover, the time of energy increase was basically the same when ST36 and ST4 were magnetically stimulated. This indicates that magnetic stimulation of ST36 and ST40 have basically the same influence on the oscillation of each frequency band. At the same time, we found that the brain PLV network was basically the same during magnetic stimulation at ST36 and ST40 (Figures 4–6). Therefore, we hypothesized that magnetic stimulation at ST36 and ST40 of the same meridians would have basically the same effect on the brain. When we carried out magnetic stimulation at ST36 and ST40, the signals are transmitted to the brain and will regulate the signals in various brain regions and frequency bands in the brain. By adjusting the signal communication between brain regions, the brain can affect the regulation of various parts of the body. Therefore, magnetic stimulation at ST36 and ST40 located in the same meridian may be able to treat similar diseases because stimulation of these two acupuncture points has similar effects on the brain.

GB37 is located on the Gallbladder Channel of Foot Shaoyang Meridian, mainly used to treat diseases related to the eyes. Based on fMRI, Wu et al. (2008), Zhang et al. (2009), and Li et al. (2019) found that acupuncture at GB37 could activate vision-related brain regions and elicit complex brain activities in the visual cortex. Therefore, GB37 may be used to treat eye diseases by stimulating visual brain regions, which is significantly different from ST36 and ST40 in activating cognitive and pain-related brain regions.

As can be seen from Table 1, the electrodes with increased energy were mainly concentrated in the central region and frontal lobe during magnetic stimulation at GB37, which was significantly different from the results of magnetic stimulation at ST36 and ST40. Moreover, the energy of some electrodes in the right parietal lobe decreased during magnetic stimulation at GB37. According to the results of time-frequency analysis of GB37 stimulation, the energy increased in the delta, theta, and alpha bands, and the time of the energy increase was different from that of ST36 and ST40. The energy of the central region electrodes mainly increased at 50–200 ms, and that of the frontal lobe electrodes mainly increased at 70–150 ms. From the aforementioned time-frequency analysis results, it can be seen that although GB37 stimulation can also produce energy changes in the central region, the time and frequency of energy changes are different compared with ST36 and ST40. In the statistics of brain functional network, it was found that GB37 stimulation was significantly different from ST36 and ST40. As shown in Figure 5, the node degree in central region of GB37 stimulation was significantly smaller (*p* < 0.01) than that of ST36 and ST40, indicating that the functional connections of central brain region of GB37 stimulation was significantly less than that of ST36 and ST40. It is consistent with the aforementioned time-frequency analysis results of the central region. The result in this paper is also consistent with the study of Barker (1991), who found that acupuncture stimulation at ST36 resulted in increased functional connectivity in the central brain region. As shown in Figure 6, compared with stimulation at ST36 and ST40, there are also significant differences (*p* < 0.01) in the functional connections between brain areas related to central region during GB37 stimulation. The differences in brain network connections related to the central region are consistent with the results of time-frequency analysis.

In addition to the central region, time-frequency analysis also found that the energy in the parietal lobe of GB37 stimulation decreased, and the energy in the frontal lobe of GB37 stimulation also changed. These results may be related to the brain network connections between brain regions. In conclusion, magnetic stimulation at ST36 and ST40 have basically the same effects on the brain both from the perspective of time-frequency analysis and brain functional network, while magnetic stimulation at GB37 has significantly different effects (*p* < 0.01) from ST36 and ST40. The main reason for the aforementioned difference is speculated to be related to the fact that ST36 and ST40 are located on the same meridian, while GB37 is located on a different meridian. Stimulation at the acupoints on the different meridians has different effects on the brain, so as to achieve the treatment of different diseases. Based on EEG and magnetic stimulation technology, this paper used short-time Fourier transform, PLV, and graph theory to analyze the time-frequency results and the brain networks of magnetic stimulation at ST36, ST40, and GB37. The time-frequency analysis results showed that the energy of left parietal lobe and right central region increased in the theta and alpha bands during magnetic stimulation at ST36 and ST40. When GB37 was magnetically stimulated, the energy did not increase in the left parietal lobe, and the energy of the frontal lobe and central area increased in the delta band. Results related to time-frequency analysis were also obtained in PLV brain network analysis. The brain network connections related to central region of GB37 stimulation were significantly different (*p* < 0.01) from that of ST36 and ST40.

From the perspective of time-frequency analysis, we can see that the frequency band adjusted by magnetic stimulation of GB37 is significantly different from that of ST36 and ST40, which indicates that magnetic stimulation of GB37 has different effects on the brain from ST36 and ST40. From the perspective of brain network, we can see that the activity degree of each brain area in magnetic stimulation of GB37 is also significantly different from that of ST36 and ST40, indicating that the brain area affected by magnetic stimulation of GB37 is also different from that of ST36 and ST40. At the same time, the brain regions where time-frequency analysis differences occurred were consistent with the brain network, which also confirmed the validity of the results of time-frequency analysis and brain network analysis. Magnetic stimulation at GB37 affects the communication between brain regions and brain regions by adjusting signals in different frequency bands, so as to regulate other organs of the body and ultimately achieve the treatment of different diseases. In the study of single acupoints, Liu et al. (2013) found that local efficiency of brain network was improved after acupuncture stimulation. Yu et al. (2018) found that clustering coefficient of brain functional network increased after acupuncture, indicating that acupuncture can improve network integration. The results of this paper show that there are certain differences in brain network characteristics when magnetic stimulation is applied to different acupoints, which indicates that different brain regions are activated at different acupoints, which may lead to different diseases.

There are two major disadvantages in this paper: (1) The EEG data we used were scalp EEG data, and all the analysis was based on the position of electrodes on the scalp. It is not known which intracranial brain regions are affected by magnetic stimulation at acupoints, so subsequent studies can use traceability analysis and use EEG and fMRI in combination. (2) We mainly analyzed the influence of magnetic stimulation on the electrical signals in the brain, but could not observe the influence of magnetic stimulation on other parts of the body and could not know how the changes of brain signals regulate the body organs. Therefore, subsequent studies can use relevant medical methods or combine with clinical analysis.

For example, Liu et al. (2012) and Chen et al. (2020) studied the effects of contralateral acupuncture of Quchi and Zusanli on regional uniformity in ischemic stroke patients with left hemiplegia, and also used fMRI research methods. Therefore, in the future, magnetic acupuncture points can be combined with the treatment of disease and fMRI to observe the help of magnetic acupuncture points in the treatment of disease and which brain regions play a role. Vestibular stimulation is a hot topic at the moment. Ko et al. (2020) used noisy vestibular stimulation in patients with bilateral vestibular dysfunction and observed EEG data. In the future, magnetic stimulation could also be combined with vestibular stimulation to see if it affects the EEG or fMRI of patients with related disorders.

This paper attempts to observe the specificity of the acupoints by studying the time-frequency results between groups of stimulation at acupoints on the same and different meridians, and tries to provide new evidence for the acupoint specificity. It is helpful to reveal the brain regulation mechanism of acupoint stimulation and has clinical application prospect in the visual evaluation of the effect of acupoint stimulation therapy.

## Data Availability Statement

The raw data supporting the conclusions of this article will be made available by the authors, without undue reservation.

## Ethics Statement

The studies involving human participants were reviewed and approved by the Biomedical Laboratory, Hebei University of Technology. The patients/participants provided their written informed consent to participate in this study.

## Author Contributions

NY and A-XW conceived and designed the experiments, analyzed the data, and wrote the manuscript. A-XW performed the experiments. All authors contributed to the article and approved the submitted version.

## Conflict of Interest

The authors declare that the research was conducted in the absence of any commercial or financial relationships that could be construed as a potential conflict of interest.

## Publisher’s Note

All claims expressed in this article are solely those of the authors and do not necessarily represent those of their affiliated organizations, or those of the publisher, the editors and the reviewers. Any product that may be evaluated in this article, or claim that may be made by its manufacturer, is not guaranteed or endorsed by the publisher.
